# Supplementary material for: Ionic Liquid-Assisted Fabrication of Bioactive Heterogeneous Magnetic Nanocatalyst with Antioxidant and Antibacterial Activities for the Synthesis of Polyhydroquinoline Derivatives
Source: Molecules. 2022 Mar 7;27(5):1748. doi: 10.3390/molecules27051748 (PMC8912081; doi:10.3390/molecules27051748)
Supplement: Supplementary file 1 [file molecules-27-01748-s001.zip › molecules-1633090-supplementary.pdf]

Ionic Liquid-assisted Fabrication of Bioactive Heterogeneous Magnetic Nanocatalyst with Antioxidant and Antibacterial Activities for the Synthesis of Polyhydroquinoline Derivatives

Shefa Mirani Nezhad[1], Ehsan Nazarzadeh Zare[1*], Azimeh Davarpanah[1], Seied Ali Pourmousavi[1], Milad Ashrafizadeh[2], Alan Prem Kumar[3,4]

[1] School of Chemistry, Damghan University, Damghan 36716-41167, Iran; shefamirani@yahoo.com (S.M.N.); a.d.13962017@gmail.com (A.D.); pourmousavi@du.ac.ir (S.A.P.)

[2] Faculty of Engineering and Natural Sciences, Sabanci University, Orta Mahalle, Üniversite Caddesi No. 27, Orhanlı, Tuzla 34956, Istanbul, Turkey; dvm.milad1994@gmail.com

[3] Cancer Science Institute of Singapore and Department of Pharmacology, Yong Loo Lin School of Medicine, National University of Singapore, Singapore 117599, Singapore; apkumar@nus.edu.sg

[4] NUS Centre for Cancer Research (N2CR), Yong Loo Lin School of Medicine, National University of Singapore, Singapore 117599, Singapore

* Correspondence: ehsan.nazarzadehzare@gmail.com or e.nazarzadeh@du.ac.ir

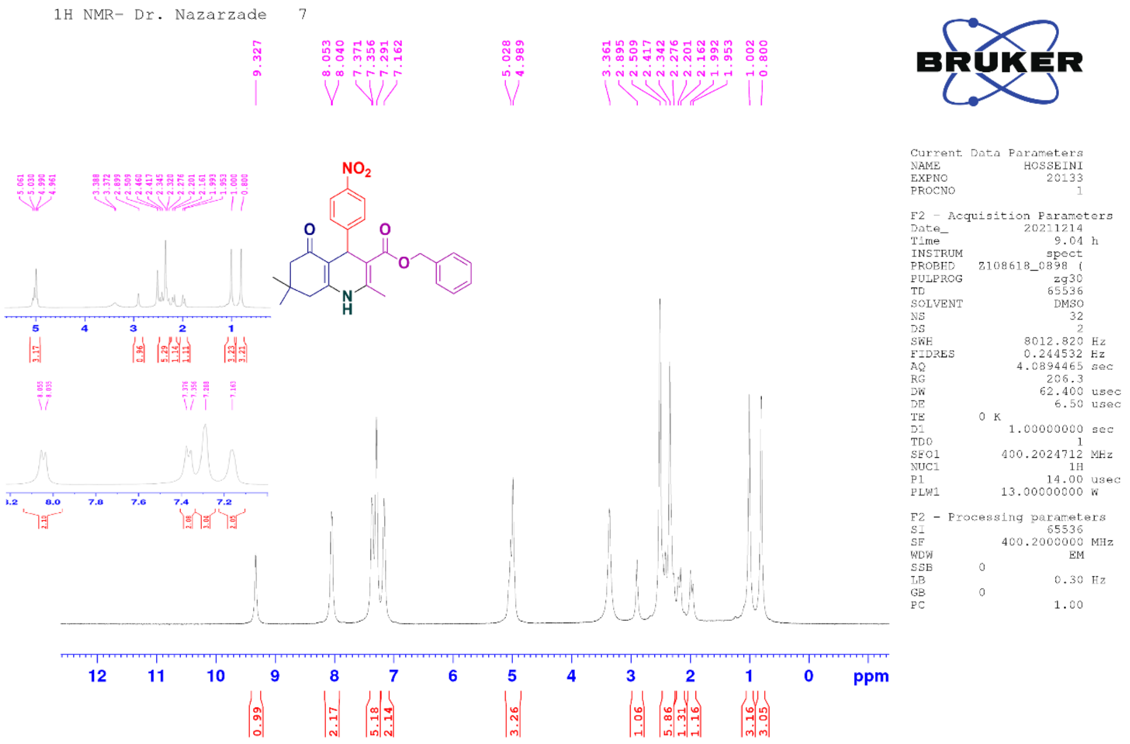

Figure S1. ^{1}H-NMR spectra of benzyl 2,7,7-trimethyl-4-(4-nitrophenyl)-5-oxo-1,4,5,6,7,8-hexahydroquinoline-3-carboxylate(**Table 5, Entry2**)

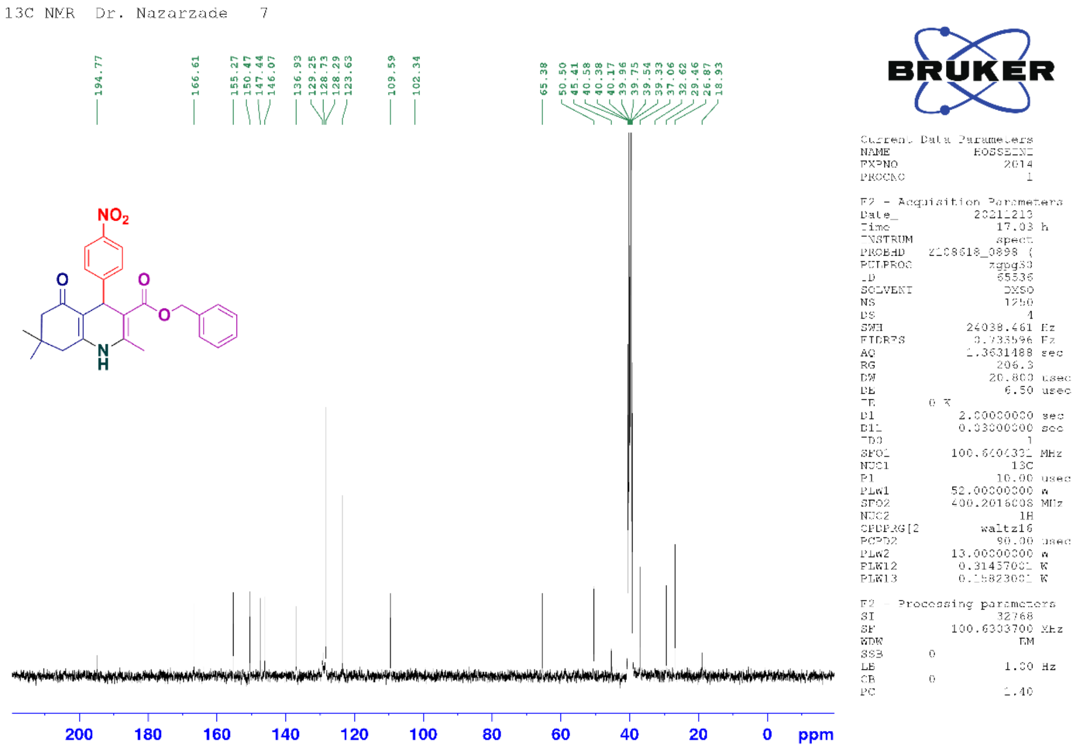

Figure S2. ^{13}C-NMR spectra of benzyl 2,7,7-trimethyl-4-(4-nitrophenyl)-5-oxo-1,4,5,6,7,8-hexahydroquinoline-3-carboxylate(**Table 5, Entry2**)

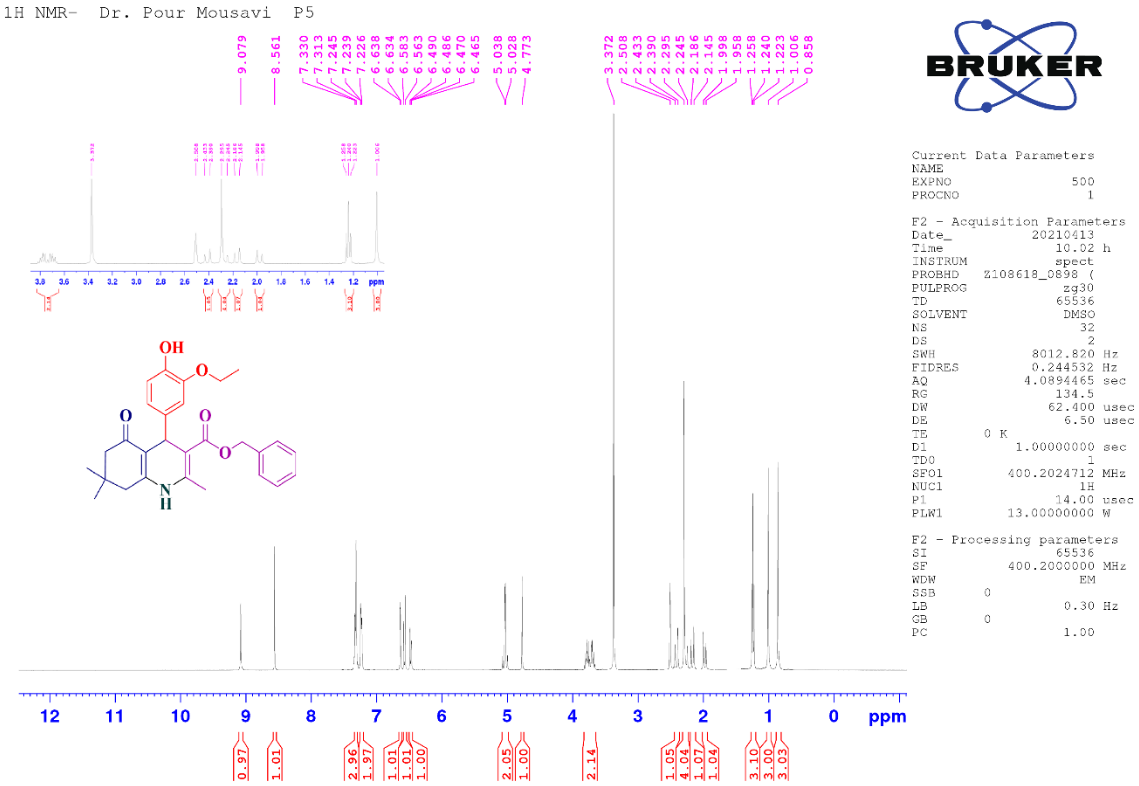

Figure S3. ^{1}H-NMR spectra of Benzyl4-(3-ethoxy-4-hydroxyphenyl)-2,7,7-trimethyl-5-oxo-1,4,5,6,7,8-hexahydroquino line -3-carboxylate (**Table 5, Entry3**)

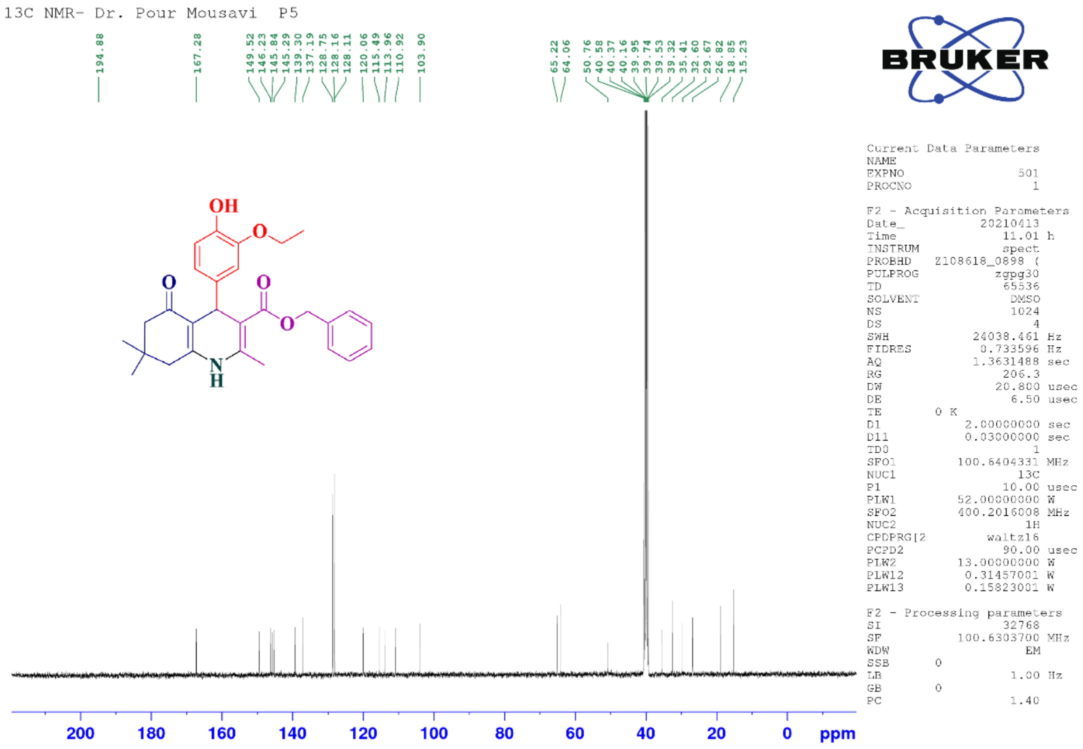

Figure S4. ^{13}C-NMR spectra of Benzyl4-(3-ethoxy-4-hydroxyphenyl)-2,7,7-trimethyl-5-oxo-1,4,5,6,7,8-hexahydroquino line -3-carboxylate (**Table 5, Entry3*)***

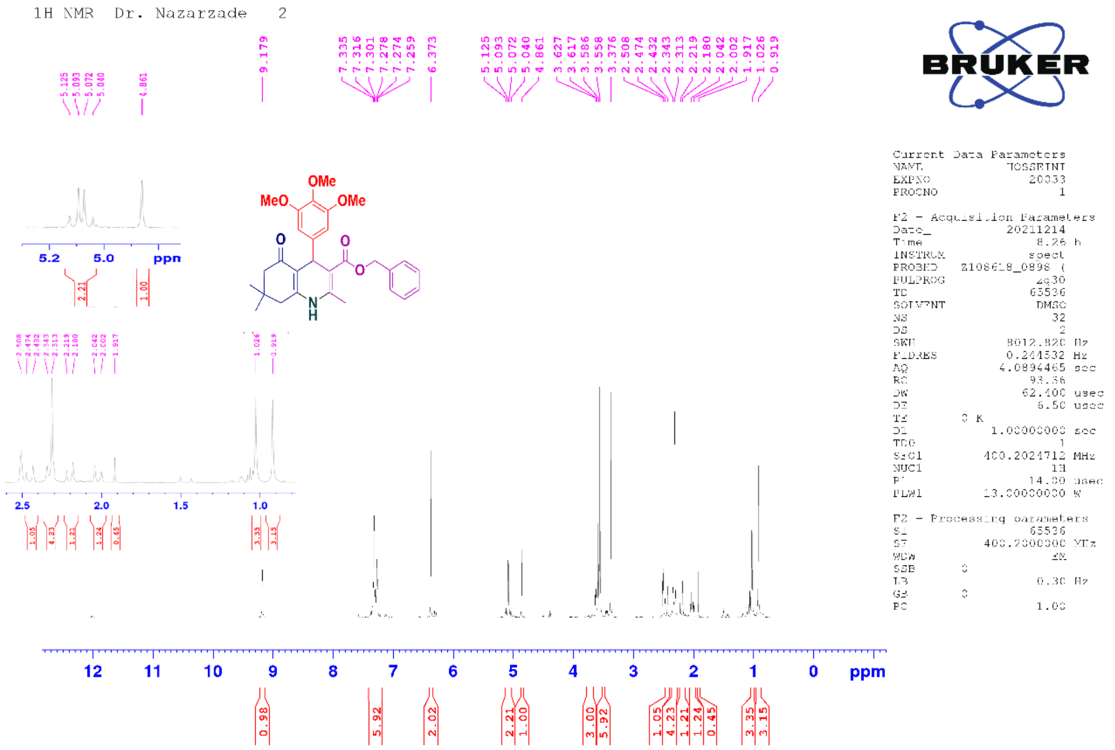

Figure S5. ^{1}H-NMR spectra of benzyl 2,7,7-trimethyl-5-oxo-4-(3,4,5-trimethoxyphenyl)-1,4,5,6,7,8-hexahydroquinoline-3-carboxylate (**Table 5, Entry 5**)

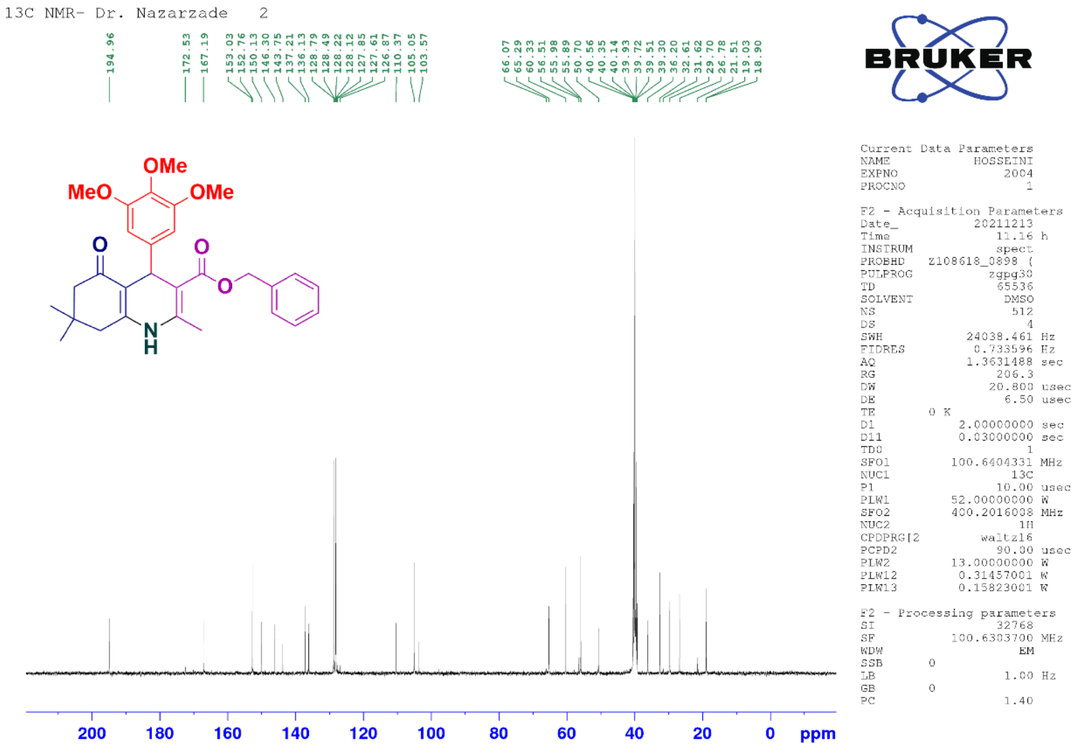

Figure S6. ^{13}C-NMR spectra of benzyl 2,7,7-trimethyl-5-oxo-4-(3,4,5-trimethoxyphenyl)-1,4,5,6,7,8-hexahydroquinoline-3-carboxylate (**Table 5, Entry 5**)

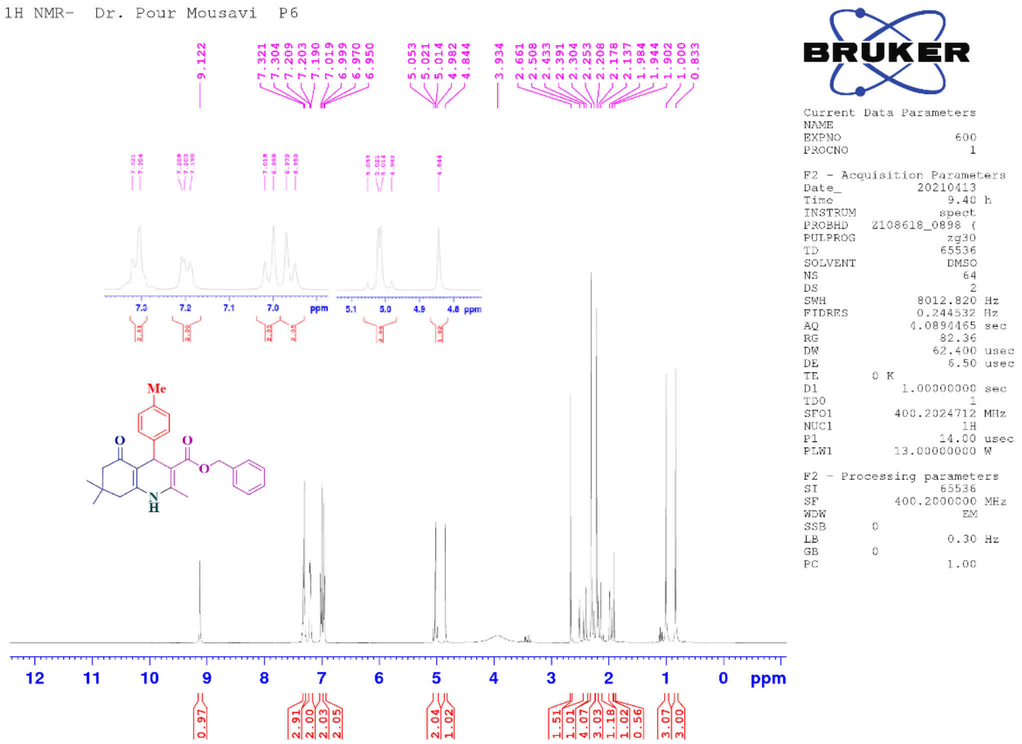

Figure S7. ^{1}H-NMR spectra of Benzyl2,7,7-trimethyl-5-oxo-4-(p-tolyl)-1,4,5,6,7,8-hexahydroquinoline-3-carboxylate (**Table 5, Entry 9**).

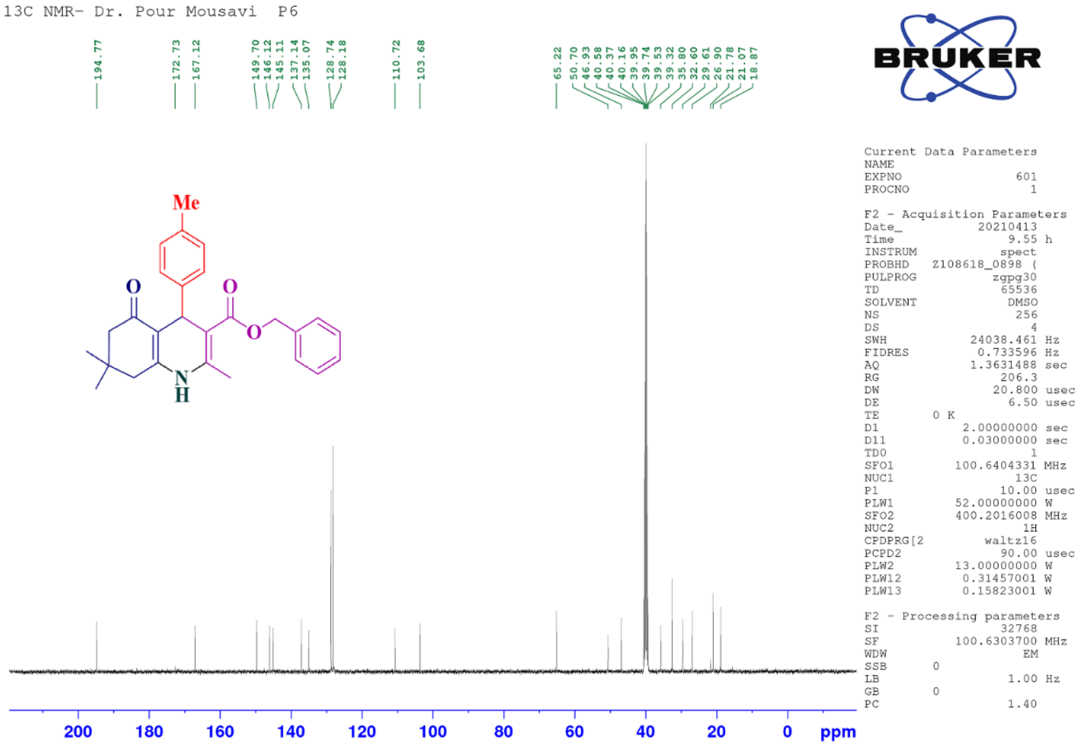

Figure S8. ^{13}C-NMR spectra of Benzyl2,7,7-trimethyl-5-oxo-4-(p-tolyl)-1,4,5,6,7,8-hexahydroquinoline-3-carboxylate (**Table 5, Entry 9**).

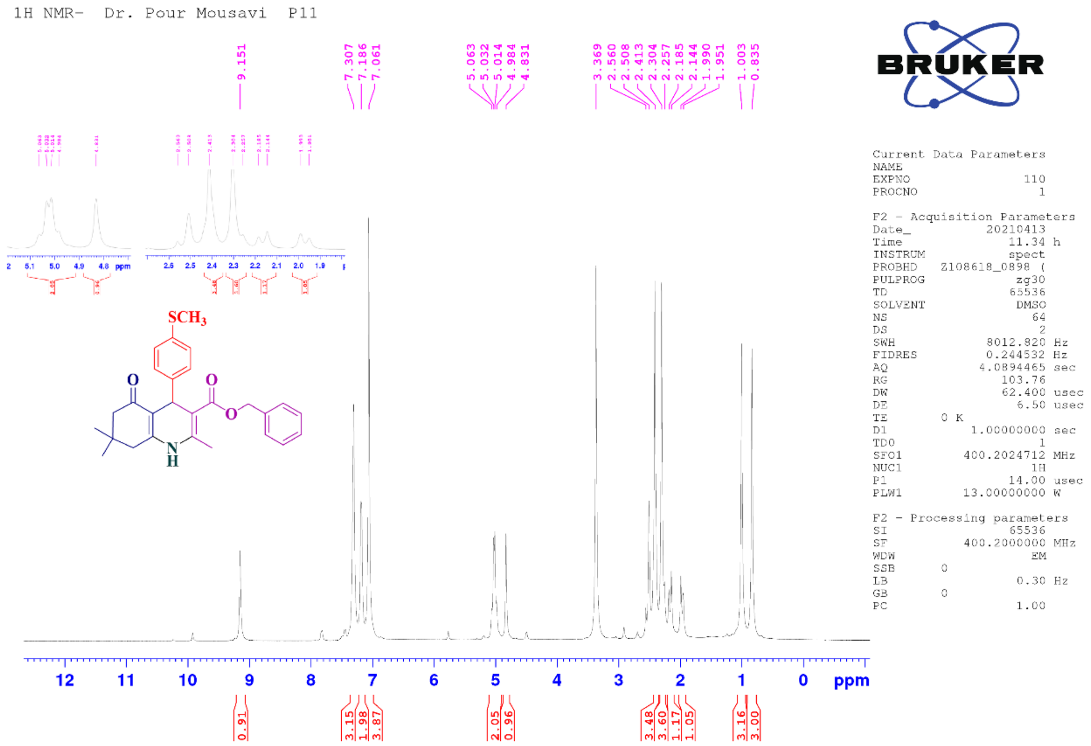

Figure S9. ^{1}H-NMR spectra of Benzyl 2,7,7-trimethyl-4-(4-(methylthio)phenyl)-5-oxo-1,4,5,6,7,8-hexahydroquinoline-3-carboxylate (**Table 5, Entry10**)

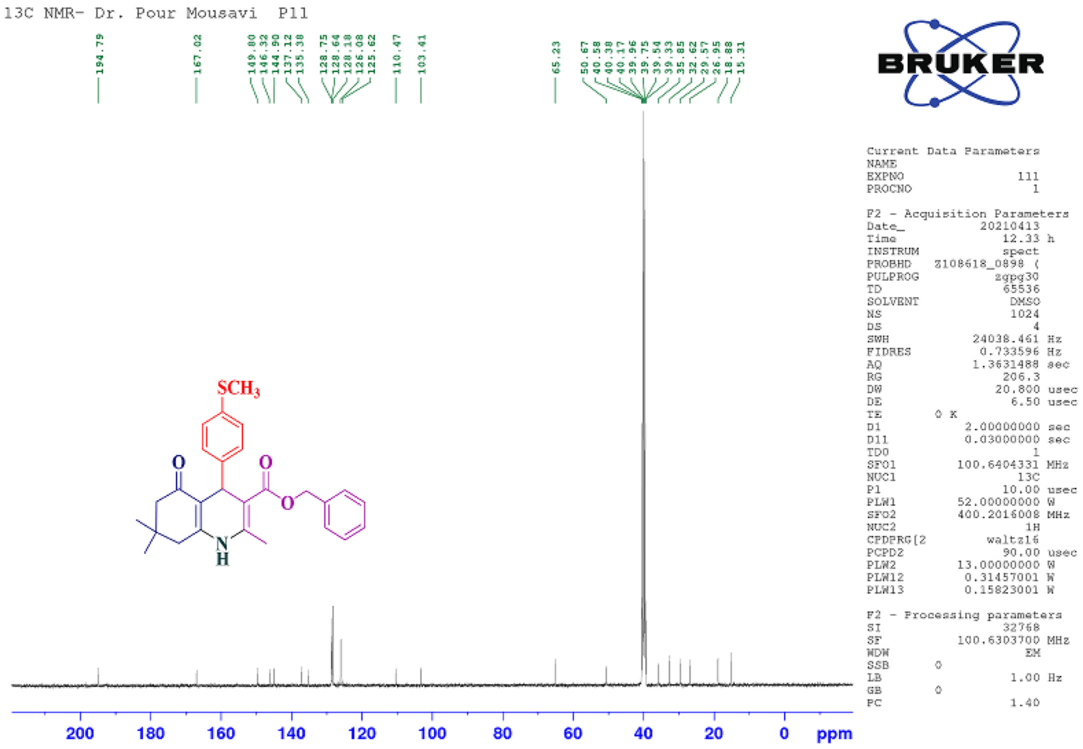

Figure S10. ^{13}C-NMR spectra of Benzyl 2,7,7-trimethyl-4-(4-(methylthio)phenyl)-5-oxo-1,4,5,6,7,8-hexahydroquinoline-3-carboxylate (**Table 5, Entry10**)

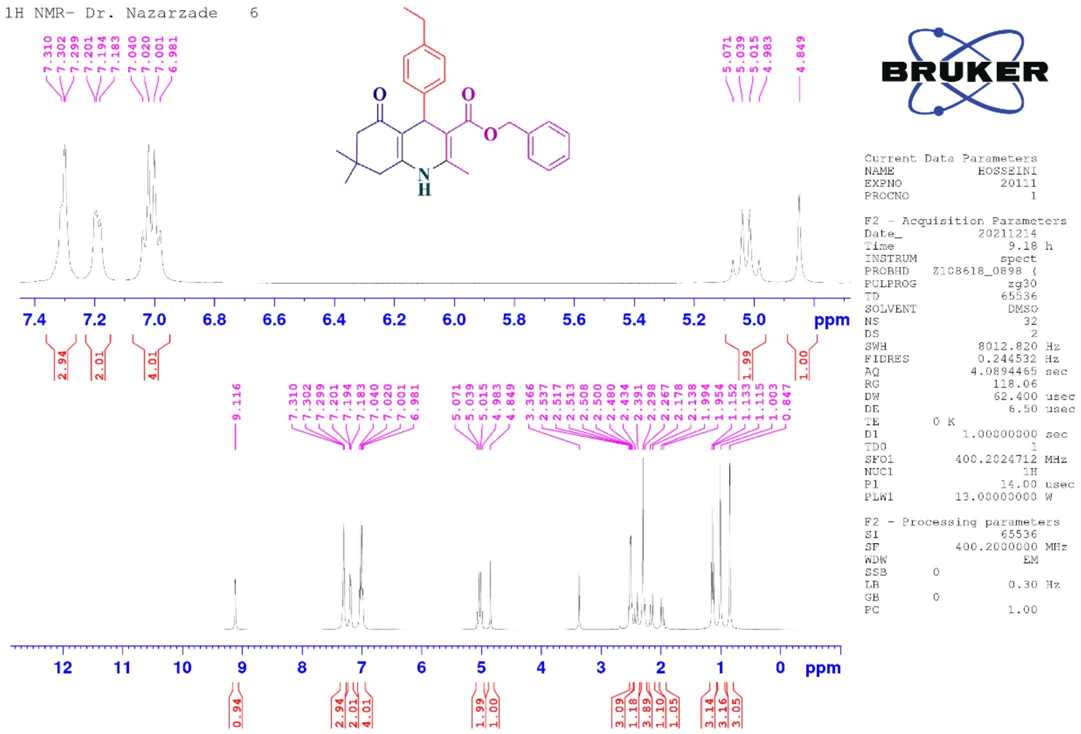

Figure S11. ^{1}H-NMR spectra of benzyl 4-(4-ethylphenyl)-2,7,7-trimethyl-5-oxo-1,4,5,6,7,8-hexahydroquinoline-3-carboxylate(**Table 5, Entry 13**)

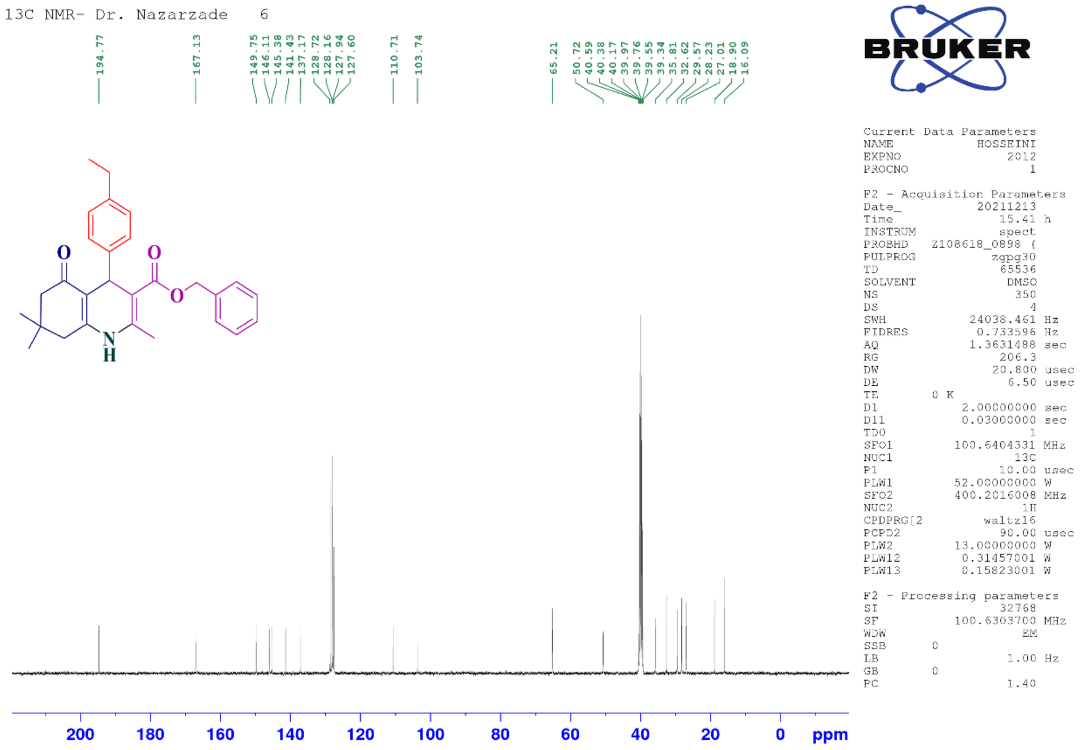

Figure S12. ^{13}C-NMR spectra of benzyl 4-(4-ethylphenyl)-2,7,7-trimethyl-5-oxo-1,4,5,6,7,8-hexahydroquinoline-3-carboxylate(**Table 5, Entry 13**)

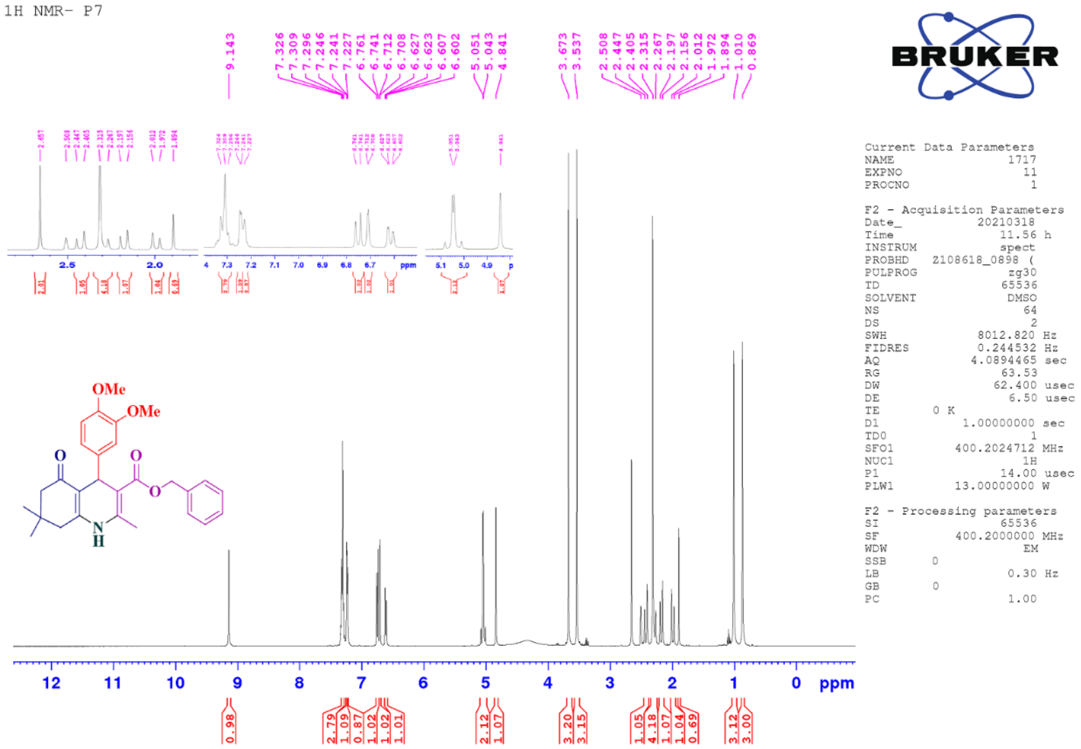

Figure S13. ¹H-NMR spectra of Benzyl 4-(3,4-dimethoxyphenyl)-2,7,7-trimethyl-5-oxo-1,4,5,6,7,8-hexahydroquinoline-3-carboxylate (**Table 5, Entry15**)

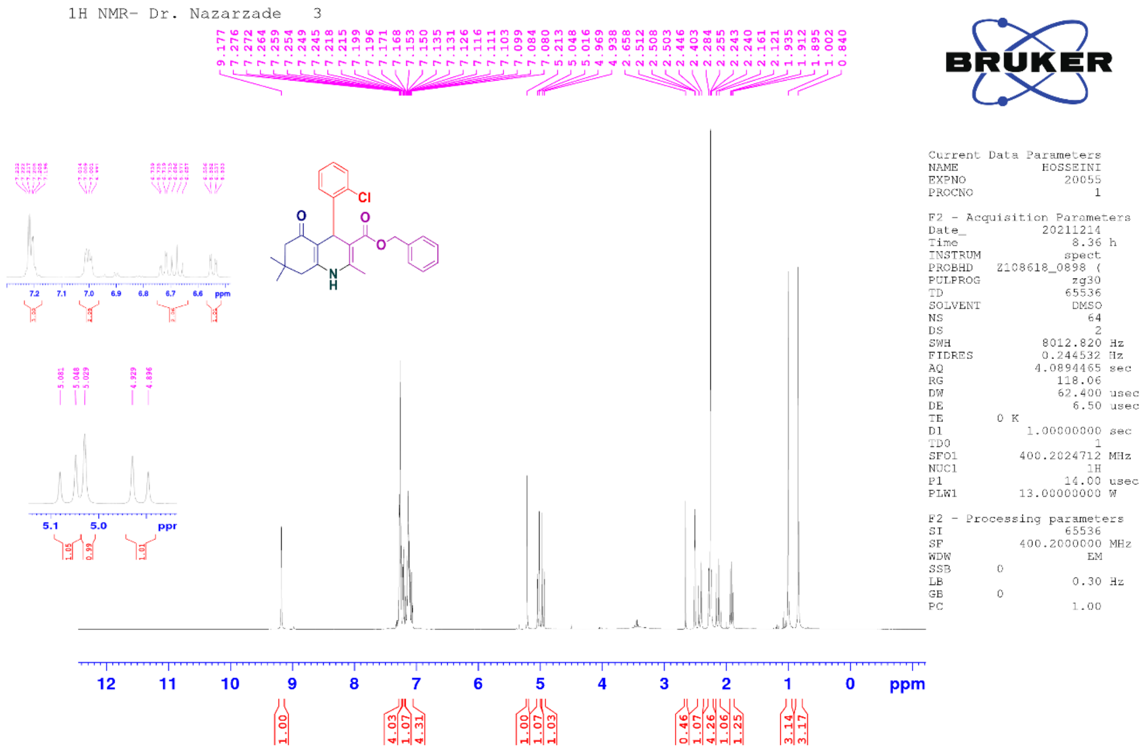

Figure S14. ¹H-NMR spectra of benzyl 4-(2-chlorophenyl)-2,7,7-trimethyl-5-oxo-1,4,5,6,7,8-hexahydroquinoline-3-carboxylate (**Table 5, Entry 18**)

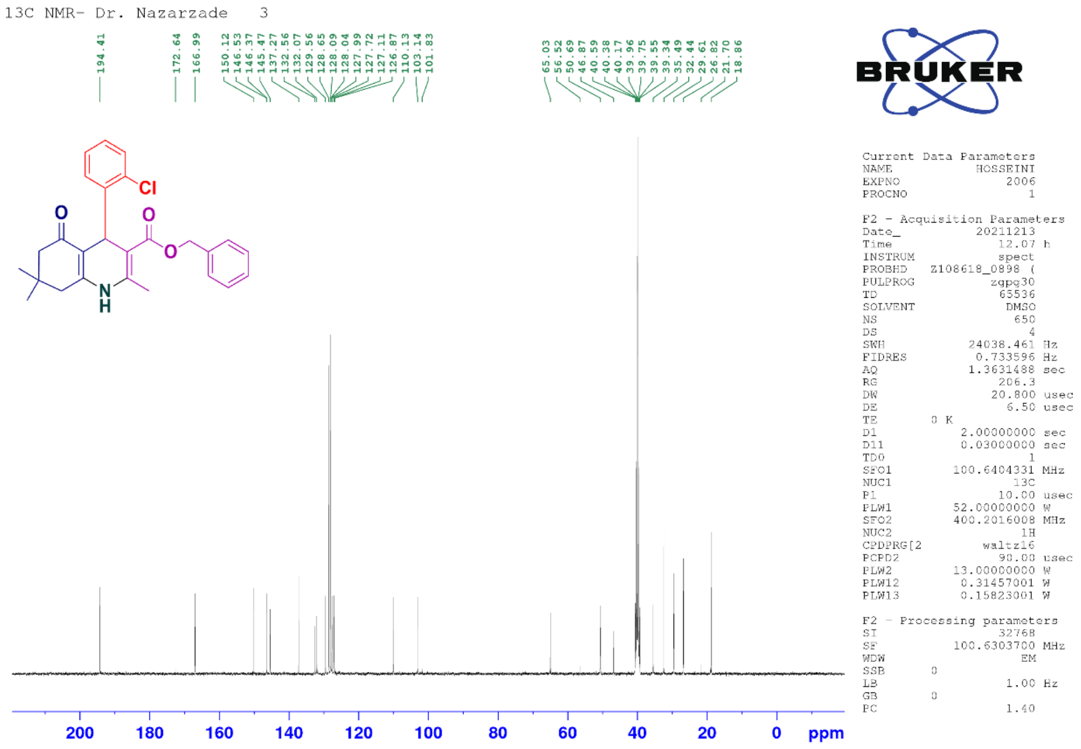

Figure S15. ^{13}C-NMR spectra of benzyl 4-(2-chlorophenyl)-2,7,7-trimethyl-5-oxo-1,4,5,6,7,8-hexahydroquinoline-3-carboxylate (**Table 5, Entry 18**)

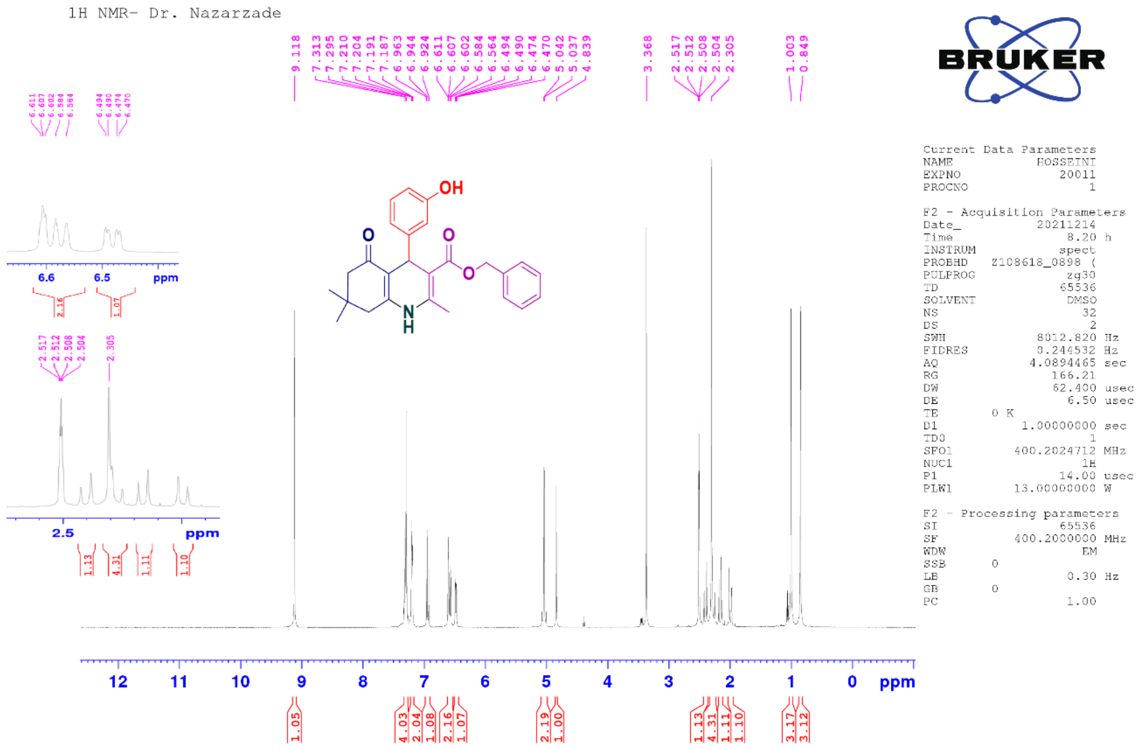

Figure S16. ^{1}H-NMR spectra of benzyl 4-(3-hydroxyphenyl)-2,7,7-trimethyl-5-oxo-1,4,5,6,7,8-hexahydroquinoline-3-carboxylate (**Table 5, Entry 19**)

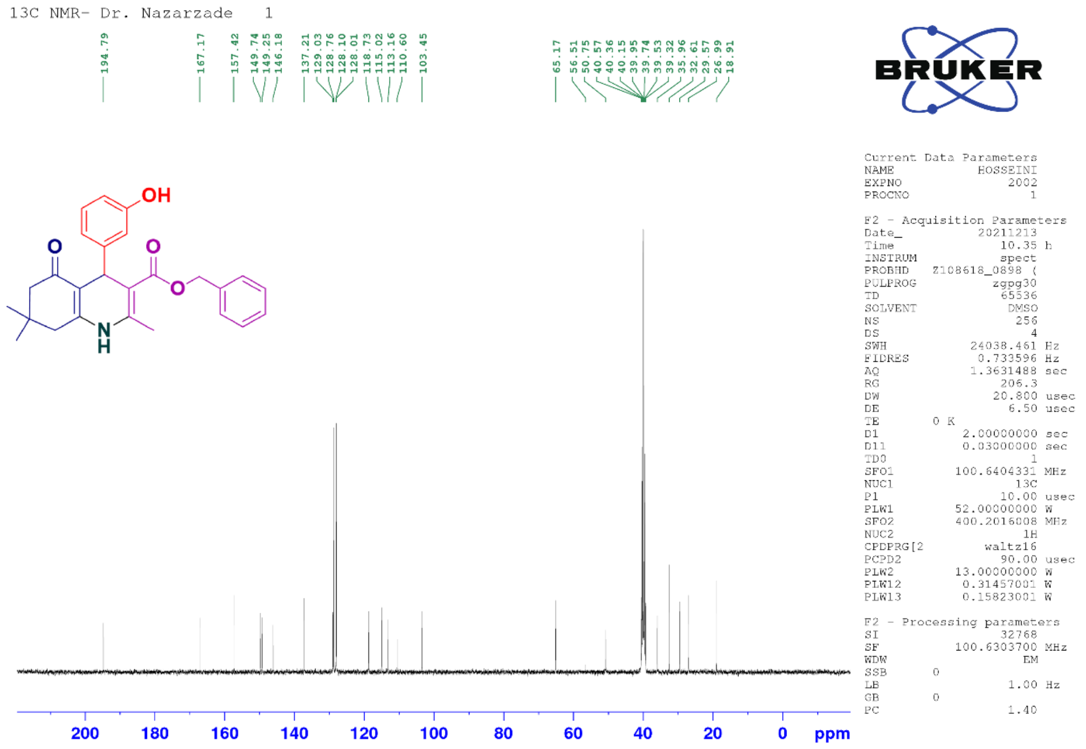

Figure S17. ^{13}C-NMR spectra of benzyl 4-(3-hydroxyphenyl)-2,7,7-trimethyl-5-oxo-1,4,5,6,7,8-hexahydroquinoline-3-carboxylate (**Table 5, Entry 19**)

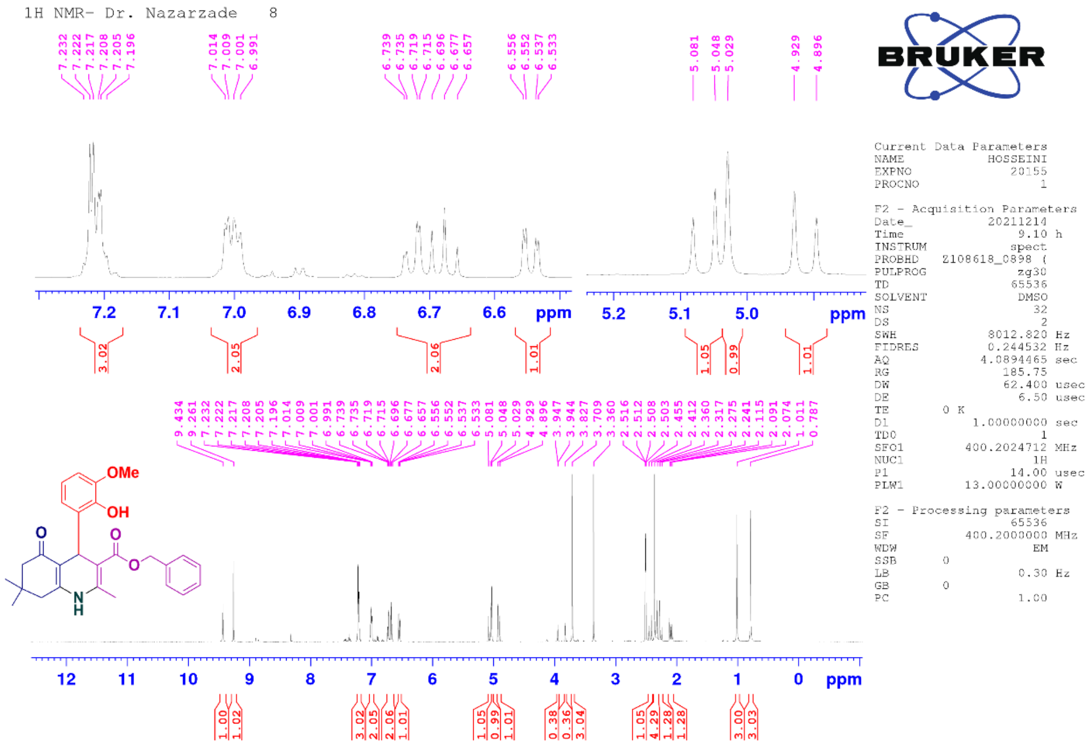

Figure S18. ^{1}H-NMR spectra of benzyl 4-(2-hydroxy-3-methoxyphenyl)-2,7,7-trimethyl-5-oxo-1,4,5,6,7,8-hexahydroquinoline-3-carboxylate(**Table 5, Entry 20**)

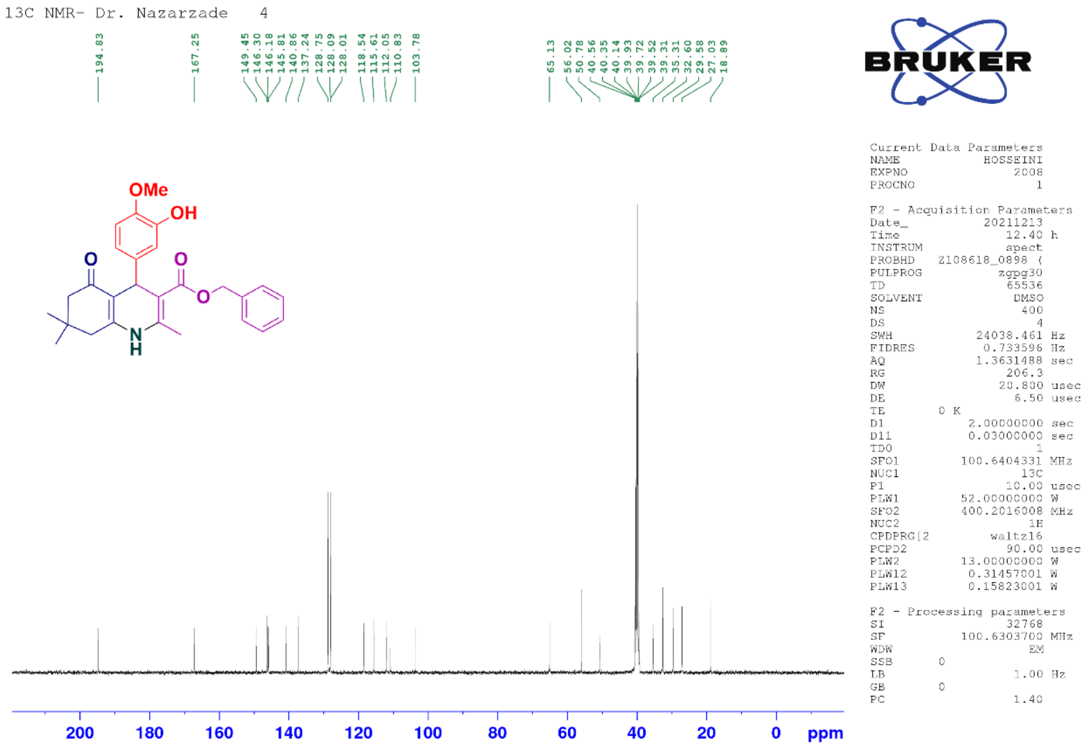

Figure S19. ^{13}C-NMR spectra of benzyl 4-(3-hydroxy-4-methoxyphenyl)-2,7,7-trimethyl-5-oxo-1,4,5,6,7,8-hexahydroquinoline-3-carboxylate(**Table 5, Entry 20**)

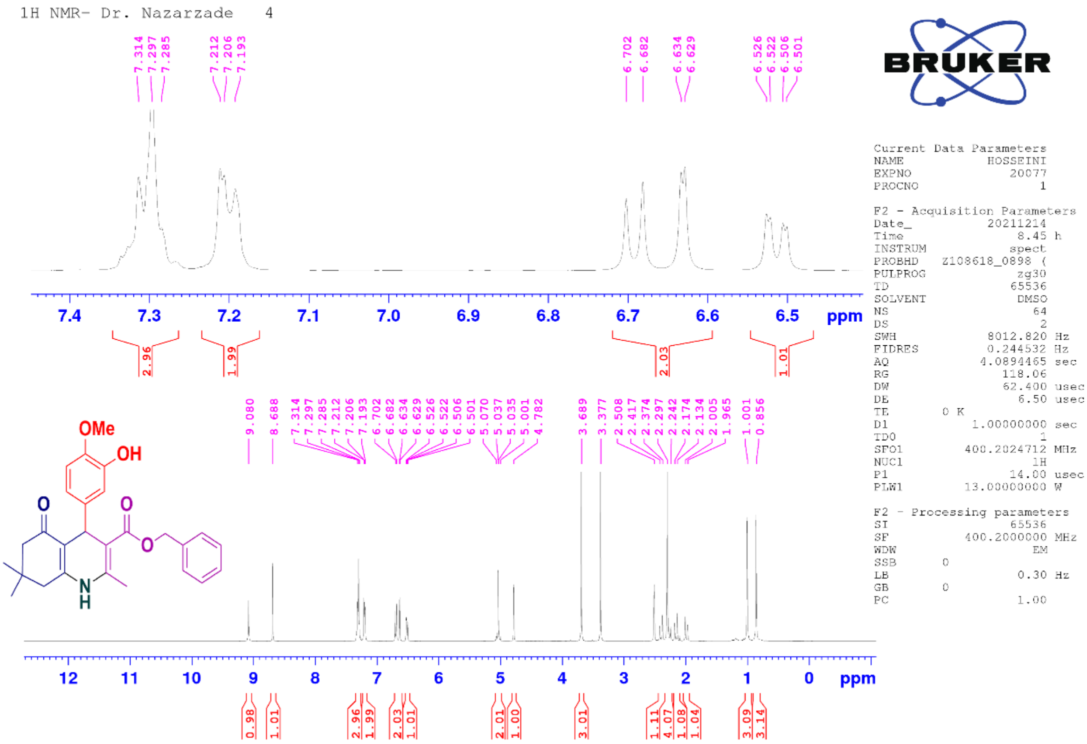

Figure S20. ^{1}H-NMR spectra of benzyl 4-(3-hydroxy-4-methoxyphenyl)-2,7,7-trimethyl-5-oxo-1,4,5,6,7,8-hexahydroquinoline-3-carboxylate(**Table 5, Entry22**)

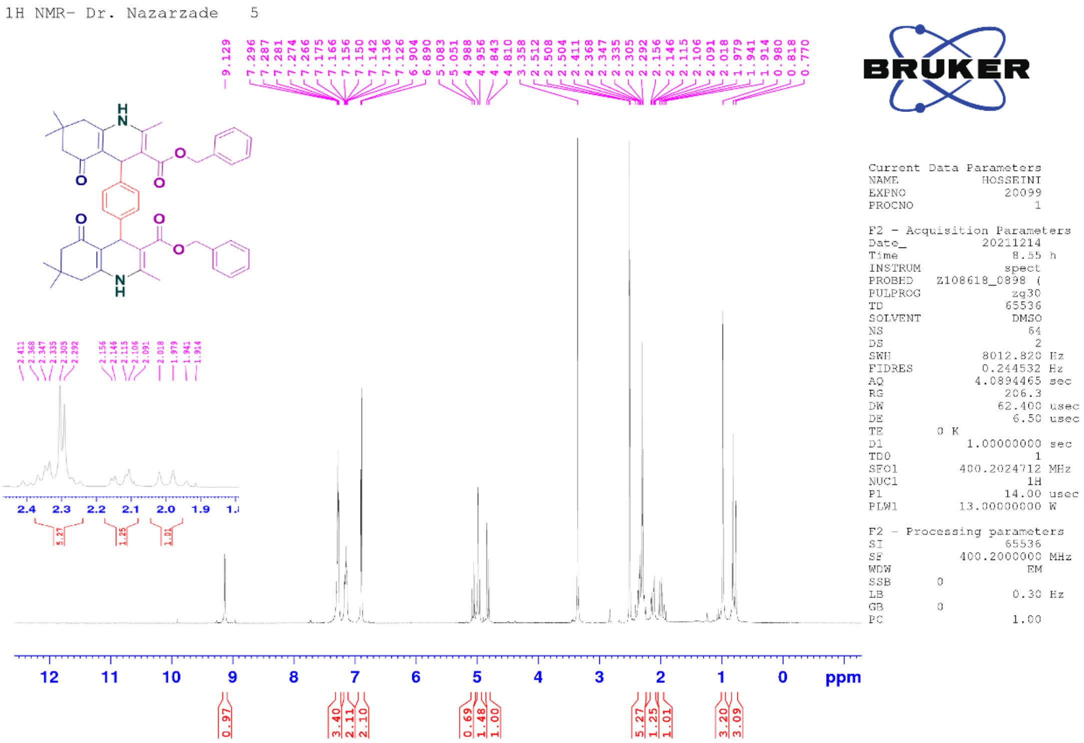

Figure S21. ^{1}H-NMR spectra of dibenzyl 4,4'-(1,4-phenylene)bis(2,7,7-trimethyl-5-oxo-1,4,5,6,7,8-hexahydroquinoline-3-carboxylate) (**Table 5, Entry23**)

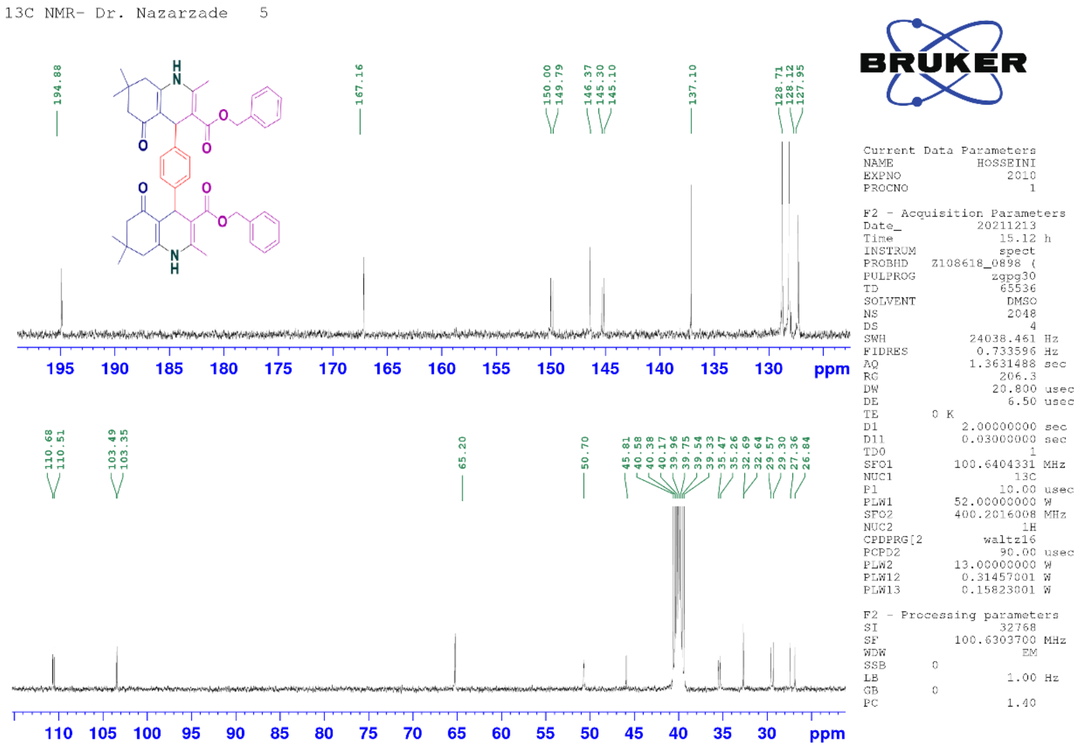

Figure S22. ^{13}C-NMR spectra of dibenzyl 4,4'-(1,4-phenylene)bis(2,7,7-trimethyl-5-oxo-1,4,5,6,7,8-hexahydroquinoline-3-carboxylate) (**Table 5, Entry 23**)

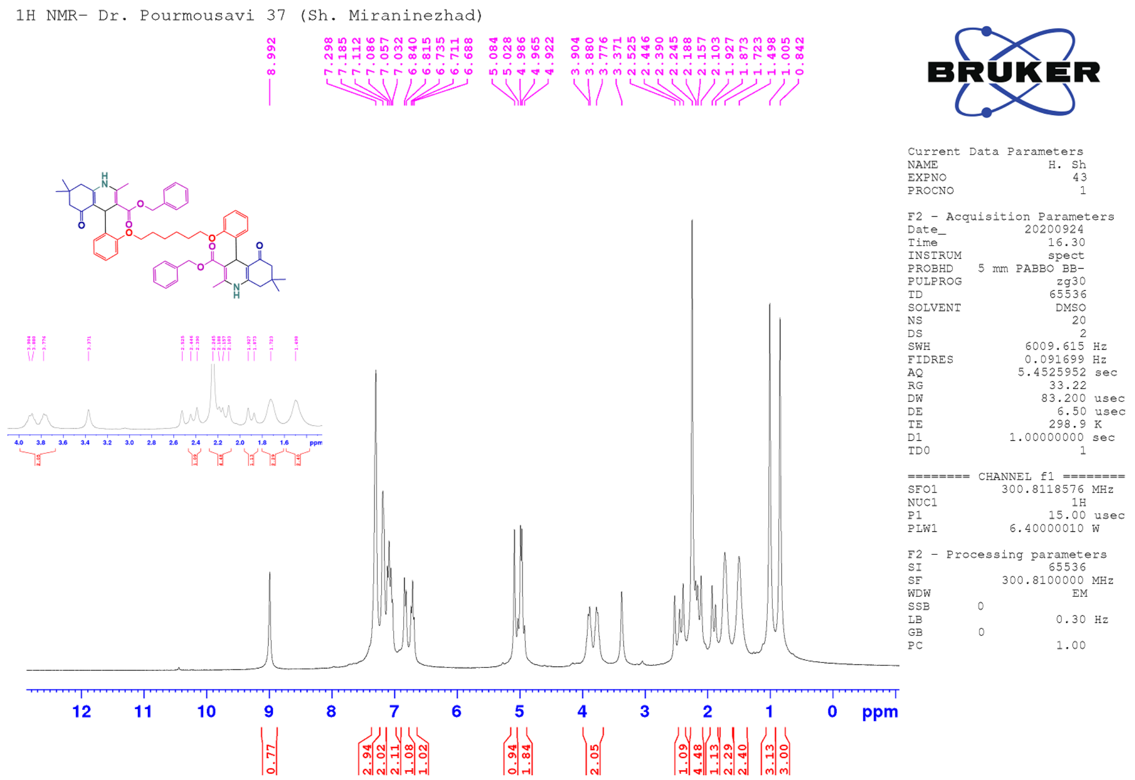

Figure S23. ^{1}H-NMR spectra of Dibenzyl 4,4'-((hexane-1,6-diylbis(oxy))bis(2,1-phenylene))bis(2,7,7-trimethyl-5-oxo-1,4,5,6,7,8-hexahydroquinoline-3-carboxylate) (**Table 5, Entry 24**)

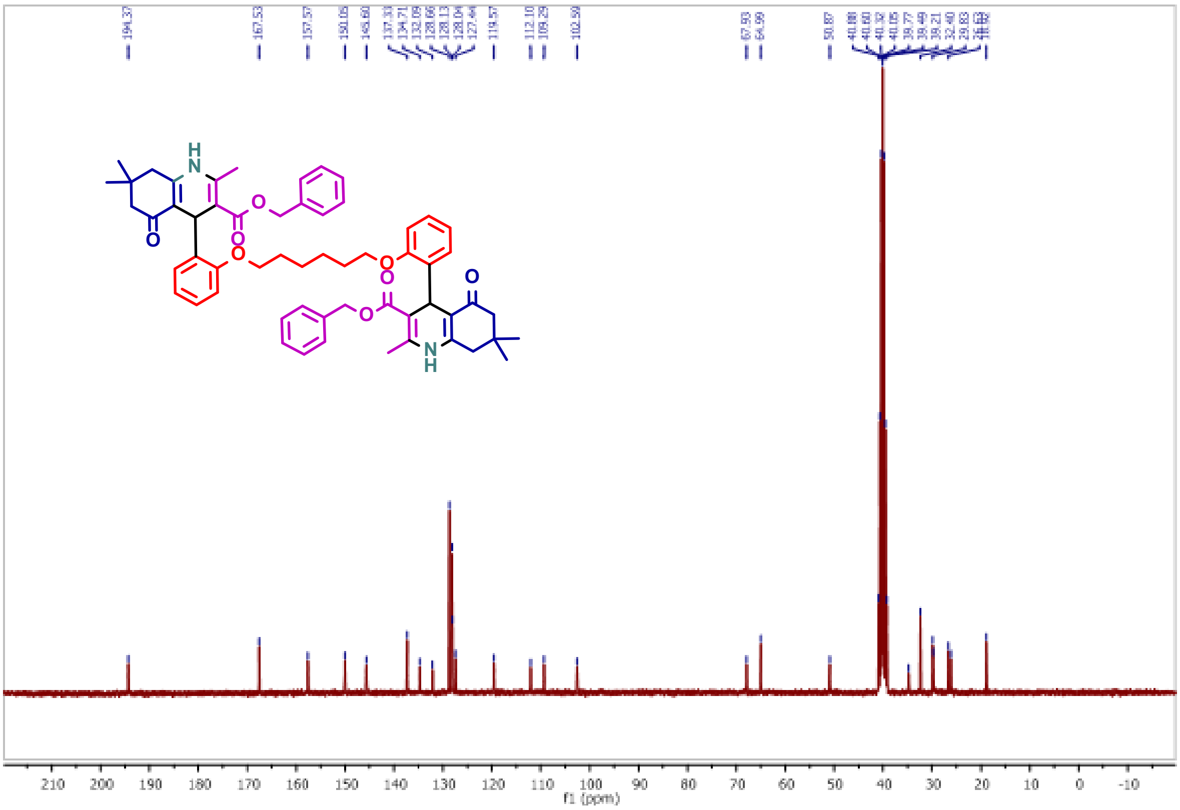

Figure S24. ^{13}C-NMR Spectra of dibenzyl 4,4'-((hexane-1,6-diylbis(oxy))bis(2,1-phenylene))bis(2,7,7-trimethyl-5-oxo-1,4,5,6,7,8-hexahydroquinoline-3-carboxylate) (**Table 3, Entry 24**)
